# Bradykinin receptors in intestinal smooth muscles and their post-receptor events related to calcium

**DOI:** 10.1155/S0962935193000432

**Published:** 1993

**Authors:** Yusuf Öztürk, V. Melih Altan, Nuray Yıdızoğlu-Arı, Orhan Altınkurt

**Affiliations:** 1 Department of Pharmacology Faculty of Pharmacy Anadolu University Eskieşhir 26470 Turkey; 2 Department of Pharmacology Faculty of Pharmacy Ankara University Ankara 06100 Turkey

## Abstract

The effects of trifluoperazine and verapamil on bradykinin- and des-Arg^9^-bradykinin induced responses of isolated rat duodenum and guinea-pig ileum were investigated to elucidate post-bradykinin receptor events. Verapamil and trifluoperazine inhibited bradykinin induced relaxations and contractions and des-Arg^9^- bradykinin induced contractions in rat duodenum. Bradykinin induced contractions of ileum were also inhibited by trifluoperazine and. verapamil. Since non-competitive affinity constants of trifluoperazine and verapamil for the relaxant responses to bradykinin in duodenum and for the contractile responses to bradykinin in ileum are different, post-bradykinin receptor events related to calcium may be different in ileum and duodenum. In addition, affinity constants of bradykinin in guinea-pig ileum and rat duodenum are also disparate suggesting the presence of different types of bradykinin B_2_ receptors in these two organs.


Mediators of Inflammation 2, 309–315 (1993)

### Bradykinin receptors in intestinal smooth muscles and their post-receptor events related to calcium

Yusuf Öztürk,[1,CA] V. Melih Altan,[2] Nuray Yıldızoğlu-Arı[2] and Orhan Altınkurt[2]

[1]Department of Pharmacology, Faculty of Pharmacy, Anadolu University, 26470 Eskişehir and [2]Department of Pharmacology, Faculty of Pharmacy, Ankara University, 06100 Ankara, Turkey

[CA] Corresponding Author

THE effects of trifluoperazine and verapamil on bradykinin- and des-Arg$^9$-bradykinin induced responses of isolated rat duodenum and guinea-pig ileum were investigated to elucidate post-bradykinin receptor events. Verapamil and trifluoperazine inhibited bradykinin induced relaxations and contractions and des-Arg$^9$-bradykinin induced contractions in rat duodenum. Bradykinin induced contractions of ileum were also inhibited by trifluoperazine and verapamil. Since non-competitive affinity constants of trifluoperazine and verapamil for the relaxant responses to bradykinin in duodenum and for the contractile responses to bradykinin in ileum are different, post-bradykinin receptor events related to calcium may be different in ileum and duodenum. In addition, affinity constants of bradykinin in guinea-pig ileum and rat duodenum are also disparate suggesting the presence of different types of bradykinin $B_2$ receptors in these two organs.

**Key words:** Bradykinin, Bradykinin receptors, Calcium, Calmodulin, Des-Arg$^9$-bradykinin, Trifluoperazine, Verapamil

### Introduction

Bradykinin and other kinins are a family of regulatory peptides involved in various pathophysiological conditions and may induce certain pharmacological actions on smooth muscles.[1,2] Among those actions, bradykinin induced relaxation of isolated rat duodenum is interesting in terms of its mode of action. Bradykinin has been reported to cause dose dependent relaxation in the isolated rat duodenum,[3,4] although some difficulties in the assessment of the relaxant responses to bradykinin have been noted.[4,5] This response to bradykinin can be changed into a biphasic effect (relaxation followed by contraction) by lowering calcium content of bathing medium.[6] However, it has been reported that high bradykinin concentration in normal medium could also elicit a biphasic effect in isolated rat duodenum.[7] As well as calcium ions, cAMP seems to be important as an intracellular mediator in the relaxant action of bradykinin.

Two kinds of receptors ($B_1$ and $B_2$) for kinins have been characterized in various smooth muscles and in the other tissues.[2] Based on the demonstration of two types of action, the existence of two receptor types for kinins in isolated rat duodenum has also been proposed.[8,9] In guinea-pig ileal muscle, there is evidence that, although the predominant receptor for bradykinin is the $B_2$ subtype, the possibility of a multiplicity of $B_2$ receptors exists in this tissue.[10,11] Recently, evidence for the multiplicity of $B_2$ in other tissues has also been obtained and the presence of $B_3$ and $B_4$ receptors for kinins has been proposed.[12–14] More recently, it has been suggested that $B_2$ receptors in rat duodenum are different from those in guinea-pig ileum.[15]

Calcium is an important intracellular mediator in the effects of various agonists. Verapamil, a calcium channel antagonist,[16] and trifluoperazine, a calmodulin antagonist,[17] are the calcium antagonists which have been widely used as pharmacological tools to investigate the physiological role of calcium ions in various tissues.

However, pharmacological properties of $B_2$ receptors in rat duodenum and in guinea-pig ileum have still not been elucidated in terms of their differences. The present study was, therefore, designed to investigate the influences of certain calcium antagonists on the bradykinin induced effects on isolated rat duodenum and guinea-pig ileum *in vitro*.

### Materials and Methods

*Experimental animals:* Adult Wistar rats (200–250 g) and guinea-pigs (300–400 g) from local strains were used. Animals were housed in a room with controlled temperature (21 ± 2°C) and humidity (65–70%). All animals were maintained in individual wire-bottom cages with *ad libitum* access to food and water. The rats and guinea-pigs used in the present study were fasted for 24 h before the experiments.

© 1993 Rapid Communications of Oxford Ltd

*Isolated rat duodenum:* Isolated rat duodenum was prepared according to a method described previously.[15] Briefly, the rats were sacrificed by a blow on the head and exsanguinated. The proximal 2 cm of duodenum was removed and suspended in a 10 ml organ bath containing Krebs' solution (pH 7.12) at 31°C, aerated with a mixture of 95% $O_2$ and 5% $CO_2$. The composition of the atropinized Krebs' solution was as follows (mM): NaCl 117.5, KCl 4.7, $CaCl_2.6H_2O$ 2.5, $KH_2PO_4$ 1.18, $MgSO_4.7H_2O$ 1.18, $NaHCO_3$ 25.0, glucose 5.5 and atropine sulphate 0.14. Isotonic recordings were made on a recording microdynamometer (Ugo Basile, No. 7050) connected to an isotonic transducer (Ugo Basile, No. 7006). All relaxations and contractions were magnified six-fold and the load on the tissue was 1.0 g. Before commencing the experiments, the isolated rat duodenum was allowed to equilibrate for 30 min. During this time, the tissue was washed every 5 min. After this initial incubation, dose–response relationships were obtained for bradykinin and des-$Arg^9$-bradykinin in the absence and in the presence of the calcium antagonists, verapamil and trifluoperazine.

*Isolated guinea-pig ileum:* Isolated guinea-pig ileum was prepared in a manner consistent with a previous method.[18] Assays were performed on the terminal ileum preparations taken from male guinea-pigs. After sacrifice, the abdomen was opened and 2–3 cm long pieces proximal to the ileocaecal sphincter were taken. Pieces of ileum were suspended in a 10 ml organ bath filled with Krebs' solution (pH 7.12) at 31°C, gassed with a mixture of 95% $O_2$ and 5% $CO_2$. For recording, tissues were connected to the isotonic transducer mentioned above. Contractions elicited by bradykinin were displayed on recording microdynamometer (Ugo Basile, No. 7050) with a five-fold magnification. The suspended ileum was maintained under a 1.0 resting tension for a 1 h stabilization period while the tissue was rinsed every 5 min. After this initial incubation, dose–response curves to bradykinin were constructed in the absence and presence of verapamil or trifluoperazine.

*Administration of doses:* Non-cumulative dose–response curves were obtained for bradykinin in the isolated rat duodenum and guinea-pig ileum. In the rat duodenum, administration of bradykinin doses ($1.0 \times 10^{-10}$ to $6.4 \times 10^{-9}$ M) were made at 5 min intervals with 30–65 s of contact time. After a 4 h incubation period, non-cumulative dose–response relationships for des-$Arg^9$-bradykinin were also obtained by administration of a dose range of $9.0 \times 10^{-8}$ to $1.4 \times 10^{-6}$ M, since a specific sensitization for contractile responses of the rat duodenum to des-$Arg^9$-bradykinin and bradykinin has been reported.[15] A 15 min dose interval for des-$Arg^9$-bradykinin was used to avoid desensitization. In isolated guinea-pig ileum, administration of bradykinin doses ($5.0 \times 10^{-9}$ to $1.6 \times 10^{-7}$ M) were made at 10 min intervals with 45–60 s of contact time. After two reproducible dose–response relationships were obtained for the agonists, the calcium antagonists, verapamil and trifluoperazine, were added to the bathing media and the tissues were incubated with these drugs for 30 min. After this incubation, the same dose–response procedures were repeated for each agonist in the presence of verapamil or trifluoperazine. In each experiment, only one concentration of an antagonist was tested against one agonist.

*Analysis of data and statistics:* Contractions and relaxations in the isolated tissues were expressed as a percentage of the corresponding maximal response to each agonist. To evaluate the relaxant and contractile effect, $pD_2$ values (apparent agonist affinity constants) of bradykinin and des-$Arg^9$-bradykinin were calculated, while $pD'_2$ values (non-competitive antagonist affinity constant) were used in the pharmacological evaluation of the antagonistic effects of trifluoperazine and verapamil.[19,20] Regression analyses were also applied in order to examine parallelism between the non-cumulative dose–response curves obtained in the absence and presence of the antagonists used.[21] Values reported represent the mean $\pm$ S.E.M. (standard error of mean) of the results of individual experiments. Significance of differences was determined by the Student's *t*-test for paired data.[22] All calculations were made by using a computer program written in BASIC language.[23]

*Drugs:* The following drugs were used: bradykinin triacetate, des-$Arg^9$-bradykinin and trifluoperazine hydrochloride, all purchased from Sigma Chemical Co. (St. Louis, USA) and verapamil hydrochloride, obtained from Knoll (Germany). Solutions of non-peptide drugs were freshly prepared in 0.9% NaCl before use. Several concentrations of each peptide solution were made immediately prior to each experiment and kept at 4°C until administration, at which time the temperature of the solution was allowed to equilibrate to 20°C. Bradykinin was dissolved in fresh saline, while des-$Arg^9$-bradykinin solution was prepared in 0.83 M acetic acid. Vehicle (acetic acid) control response was checked in isolated rat duodenum. Acetic acid in the dilutions used did not affect the tissue responses. All drugs were added to the organ bath in a volume not exceeding 0.05 ml.

### Results

*Isolated rat duodenum:* In isolated rat duodenum, bradykinin ($1.0 \times 10^{-10}$ to $6.4 \times 10^{-9}$ M) (Fig. 1A)

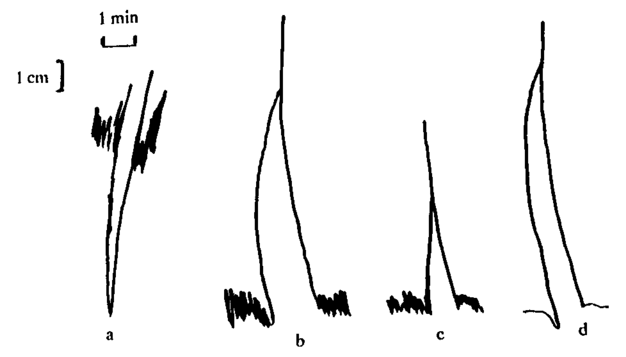

FIG. 1. Typical responses to bradykinin and des-Arg$^9$-bradykinin of isolated guinea-pig ileum and rat duodenum. a, relaxant effect of bradykinin ($3.2 \times 10^{-9}$ M); b, contractile effect of bradykinin ($1.6 \times 10^{-7}$ M); c, contractile effect of des-Arg$^9$-bradykinin ($3.2 \times 10^{-7}$ M) on the rat duodenum; d, contractile effect of bradykinin ($1.7 \times 10^{-7}$ M) on the guinea-pig ileum.

and des-Arg$^9$-bradykinin ($9.0 \times 10^{-7}$ to $6.4 \times 10^{-6}$ M) (Fig. 1C) elicited dose dependent relaxations and contractions, respectively. Bradykinin also induced a dose dependent contractile effect on the rat duodenum (Fig. 1B). The dose cycles used did not cause any desensitization to either bradykinin or des-Arg$^9$-bradykinin. $pD_2$ values for the relaxant and contractile effects of bradykinin and des-Arg$^9$-bradykinin are listed in Table 1. Bradykinin induced relaxations and contractions and des-Arg$^9$-bradykinin induced contractions in the rat duodenum were antagonized by trifluoperazine and verapamil, non-competitively (Figs. 2 and 3). $pD'_2$ values for the dose dependent antagonistic effects of trifluoperazine and verapamil are shown in Table 2.

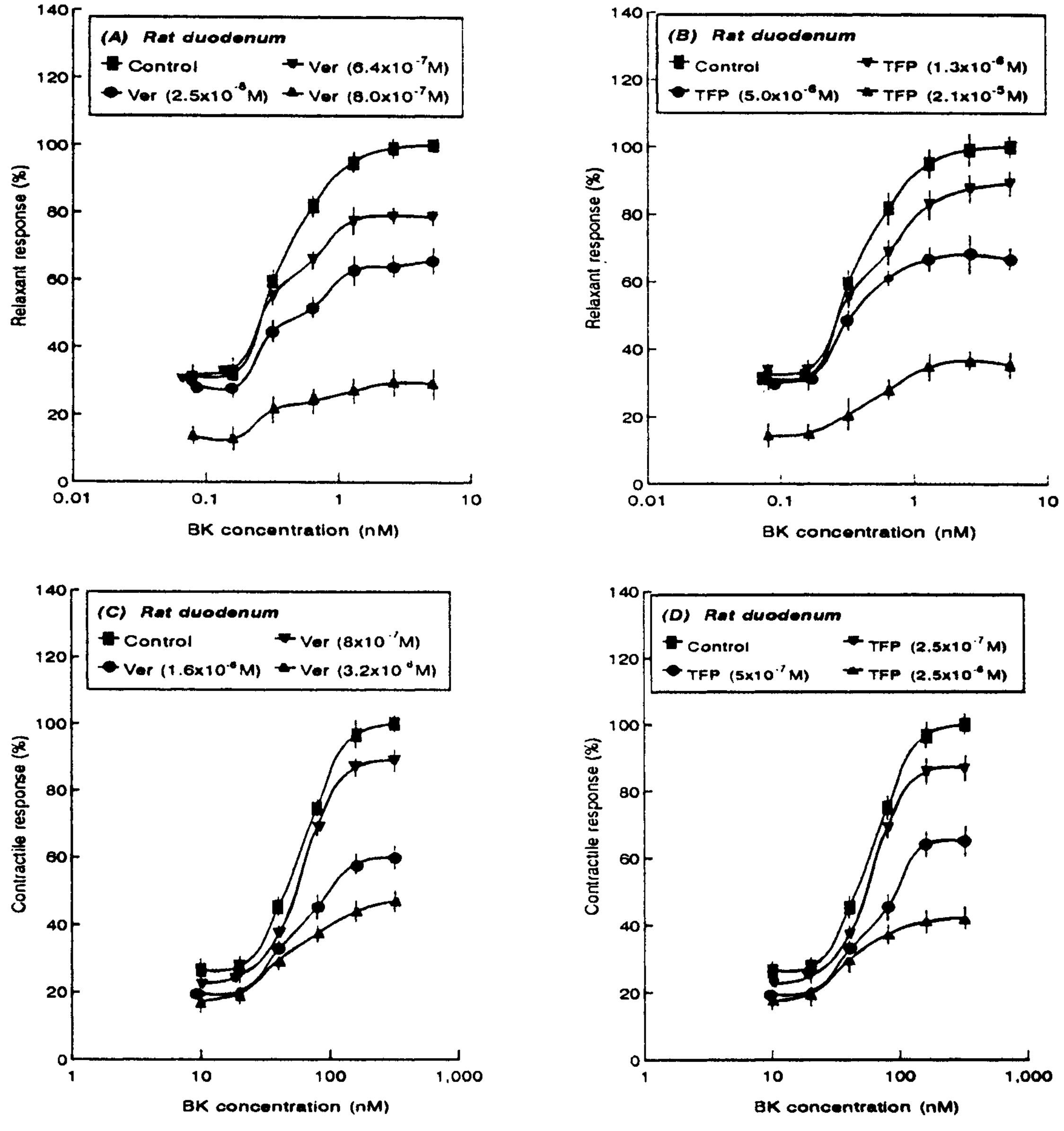

FIG. 2. Inhibitory effects of verapamil (A,C) and trifluoperazine (B,D) on the relaxant (A,B) and contractile (C,D) responses of isolated rat duodenum to bradykinin. BK, bradykinin; Ver, verapamil; TFP, trifluoperazine; vertical bars indicate standard error of mean (±S.E.M.).

**Table 1.** Apparent affinity constants ($pD_2$ values) for the effects of bradykinin and des-Arg[9]-bradykinin in the isolated rat duodenum and guinea-pig ileum

| Agonist | Rat duodenum | Guinea-pig ileum |
|---|---|---|
| Bradykinin | | |
| Relaxant response | $9.83 \pm 0.02$ ($n = 10$) | N.A.* |
| Contractile response | $7.30 \pm 0.05$** ($n = 10$) | $7.50 \pm 0.01$*** ($n = 20$) |
| Des-Arg[9]-bradykinin | $6.59 \pm 0.07$ ($n = 10$) | N.A.* |

Values represent mean (±S.E.M.) of individual results. The values in the parentheses indicate the number of experiments.
* Bradykinin and des-Arg[9]-bradykinin did not elicit reproducible relaxations of rat duodenum and contractions of guinea-pig ileum, respectively. **$p < 0.001$ and ***$p < 0.005$ significances relative to the relaxant response of rat duodenum induced by bradykinin, respectively.

*Isolated guinea-pig ileum:* Bradykinin ($2.5 \times 10^{-9}$ to $1.7 \times 10^{-7}$ M) caused dose dependent and slow onset contractions of the isolated guinea-pig ileum (Fig. 1D). No desensitization was observed for this effect with the dose cycle used in this study. The $pD_2$ value for the contractile effect of bradykinin on the guinea-pig ileum is given in Table 1. Trifluoperazine and verapamil inhibited bradykinin induced contractions of isolated guinea-pig ileum in a non-competitive manner (Fig. 4). The inhibitory effects of trifluoperazine and verapamil were also dose dependent in nature. $pD'_2$ values concerning their inhibitory effects are summarized in Table 2.

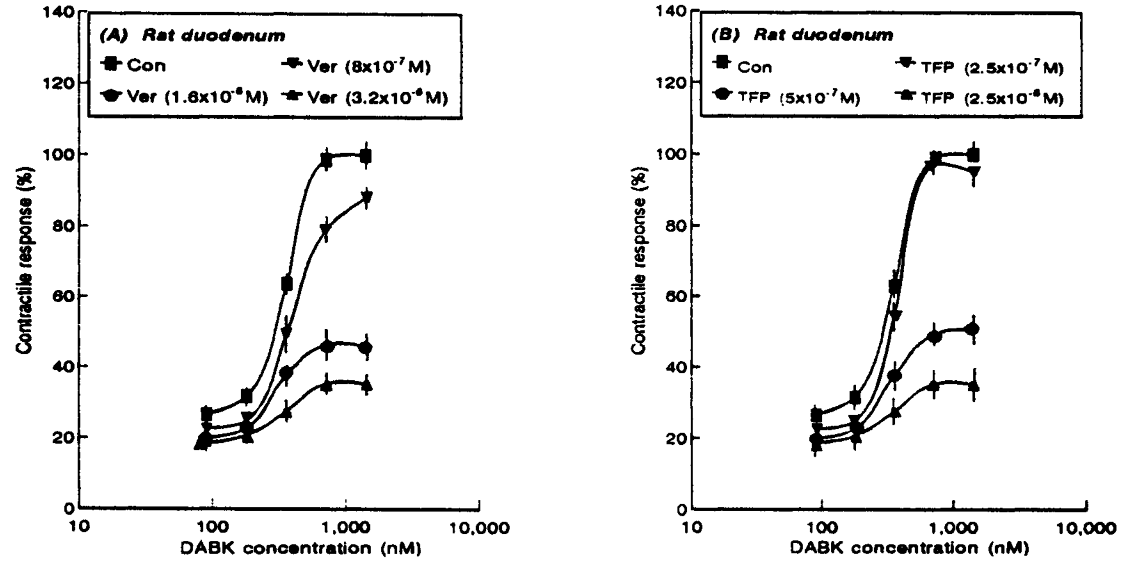

FIG. 3. Inhibitory effects of verapamil (A) and trifluoperazine (B) on the contractile responses of isolated rat duodenum to des-Arg[9]-bradykinin. DABK, des-Arg[9]-bradykinin; Ver, verapamil; TFP, trifluoperazine; vertical bars indicate standard error of mean (±S.E.M.).

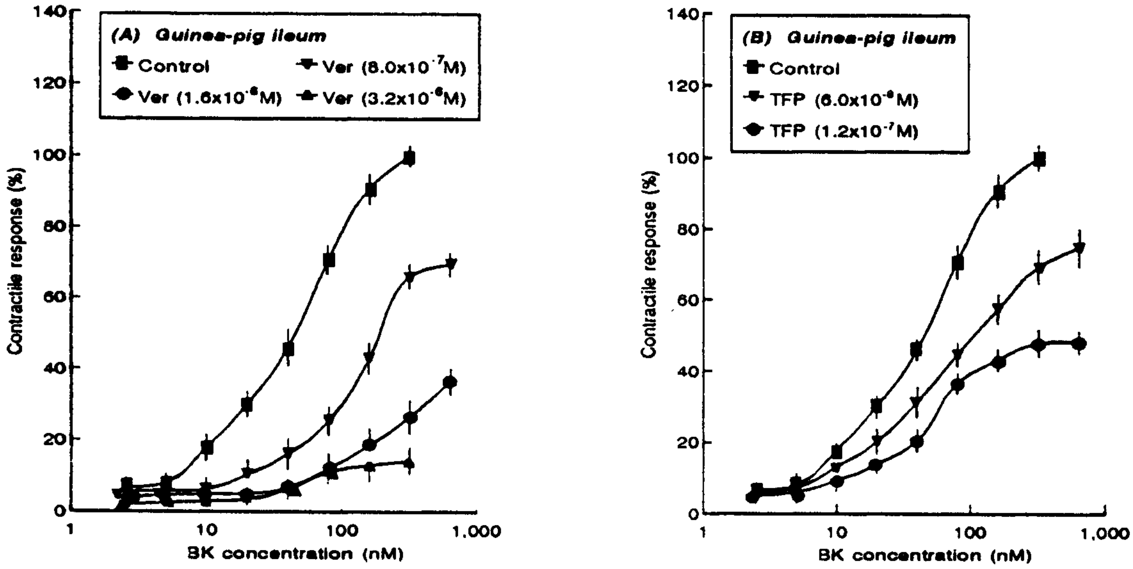

FIG. 4. Inhibitory effects of verapamil (A) and trifluoperazine (B) on the contractile responses of isolated guinea-pig ileum to bradykinin. BK, bradykinin; Ver, verapamil; TFP, trifluoperazine; vertical bars indicate standard error of mean (±S.E.M.).

**Table 2.** Non-competitive affinity constants ($pD'_2$ values) for the antagonistic effects of trifluoperazine and verapamil on bradykinin and des-Arg[9]-bradykinin induced relaxations and contractions in the isolated rat duodenum and guinea-pig ileum

Agonist Trifluoperazine Verapamil*

| Agonist | Trifluoperazine | Verapamil* |
|---|---|---|
| Isolated rat duodenum | | |
| Bradykinin (Relaxant R) | 4.99 ± 0.02** ($n = 7$) | 6.48 ± 0.07 ($n = 8$) |
| Bradykinin (Contractile R) | 6.87 ± 0.08 ($n = 8$) | 6.55 ± 0.05 ($n = 7$) |
| Des-Arg[9]-bradykinin | 6.90 ± 0.02 ($n = 8$) | 6.40 ± 0.06 ($n = 7$) |
| Isolated guinea-pig ileum | | |
| Bradykinin | 6.96 ± 0.04 ($n = 9$) | 6.44 ± 0.09 ($n = 7$) |

Values represent mean (±S.E.M.) of individual results. The values in the parentheses indicate the number of experiments. *No significant differences between the $pD'_2$ values ($p < 0.1$). **$p < 0.005$ relative to the other $pD'_2$ values.

### Discussion

This study shows that the mechanisms of the contractile and relaxant effects of bradykinin in isolated rat duodenum and guinea-pig ileum are different in regard to their dependency on cellular calcium metabolism. Isolated guinea-pig ileum is the most extensively investigated smooth muscle in terms of bradykinin effects. It has been long known that bradykinin produces a slow biphasic contraction in isolated guinea-pig ileum, contrasting with the quick, almost instantaneous effects elicited by histamine and acetylcholine.[24,25] A relaxant effect of bradykinin has been also reported in the isolated guinea-pig ileum precontracted with acetylcholine. This effect of bradykinin in guinea-pig ileum has been found to be inhibited by phentolamine[26] which has been demonstrated to have calcium antagonistic properties.[27] Attempts have been made since the late 1970s to classify the kinin receptors in the guinea-pig ileum as well as in the other tissues from certain animal species. According to the current classification, $B_2$ receptors are the predominant receptors for the effect of bradykinin in the guinea-pig ileum, whereas $B_1$ receptors seem to be absent in this tissue.[2,11] On the other hand, it has been suggested that bradykinin induced contraction and relaxation of rat duodenum are mediated by $B_1$ and $B_2$ receptors, respectively.[8,9] Des-Arg[9]-bradykinin is also effective in the isolated rat duodenum indicating the presence of $B_1$ receptors. In recent years, the evidence for the multiplicity of $B_2$ receptors has accumulated from studies using isolated gastrointestinal preparations as well as other tissues.[10,12] In fact, the existence of two more receptors ($B_3$ and $B_4$) in various tissues has been proposed.[13,14,28] Furthermore, it has been suggested that $B_2$ receptors in the rat duodenum might be different from those in guinea-pig ileum.[15] Since the $pD_2$ value for the contractile effect of bradykinin in isolated guinea-pig ileum was found to be different from that for the relaxant effect of bradykinin in the isolated rat duodenum (Table 1), it might be thought that $B_2$ receptors are different in these two tissues.

From the findings obtained in this study, post-receptor regulatory events also seem to be different in isolated guinea-pig ileum and rat duodenum. Calcium ions are important in the relaxant and contractile effects of bradykinin and des-Arg[9]-bradykinin in these gastrointestinal smooth muscles. Calcium has been known to play an important role in the signal transduction mechanisms for the bradykinin effects in various tissues.[11,14,28] Contractile responses of guinea-pig ileum and rat duodenum to bradykinin and des-Arg[9]-bradykinin were inhibited by verapamil and trifluoperazine. Verapamil is known as a calcium entry blocking agent in the concentration range used by us.[16] This implies that a calcium flux via voltage dependent channels from the extracellular space towards the cytoplasm contribute to the bradykinin- and des-Arg[9]-bradykinin induced contraction. Intracellular calcium may also play a role in the contraction caused by bradykinin as observed in various smooth muscles.[14,28,29] In that case, the formation of inositol phosphates, in particular inositol 1,4,5-triphosphate, on stimulation of bradykinin sensitive receptors is thought to release calcium from the endoplasmic reticulum to trigger the contractile elements. This calcium compartment is most likely filled with cytoplasmic reticulum by a calmodulin dependent calcium pump.[30–32] Thus, trifluoperazine induced inhibition of this process which stores calcium by interfering with calcium pumping, results in a reduced calcium release upon bradykinin $B_2$ receptor stimulation, since trifluoperazine is reported to inhibit calmodulin in the concentration range used by us.[17] Therefore, both influx of the extracellular calcium and release of the intracellular calcium seem to play an important role in the bradykinin induced contraction of isolated guinea-pig ileum via $B_2$ receptors. The mechanism for the contractile effect of des-Arg[9]-bradykinin in the isolated rat duodenum might occur in a similar manner, but the receptors involved in the calcium dependent contractile effect of des-Arg[9]-bradykinin are $B_1$ type receptors for kinins.

The inhibitory effects of verapamil may not be related to their nonspecific effects. Verapamil, but not nifedipine, have been reported to inhibit calmodulin stimulated $Ca^{2+}$–$Mg^{2+}$-ATPase in the concentrations higher than $10^{-3}$ M.[33,34] However, such a mechanism seems unlikely in the antagonistic action of verapamil against bradykinin and des-Arg$^9$-bradykinin, because the concentration required for this antagonism is approximately 100 times lower than that required for the $Ca^{2+}$–$Mg^{2+}$-ATPase inhibition. Phenothiazines including trifluoperazine possess well-known dopamine antagonistic[35] and anticholinergic[36] properties, especially in the central nervous system, which seem to be responsible for their antipsychotic activities. This effect may occur only in the central nervous system and at a plasma concentration as low as 30 ng/ml which is also decreased during the passage through the blood–brain barrier.[37] Therefore, these two mechanismsseem unlikely in the inhibitory activity of trifluoperazine against bradykinin and des-Arg$^9$-bradykinin.

On the other hand, adenylate cyclase activation, and the consequent increase in cAMP are involved in the relaxant effect of bradykinin on isolated rat duodenum[4] as well in its effect in fibroblasts.[38] In the authors' laboratory, it was also observed that nicotinic acid, an adenylate cyclase inhibitor,[39] causes a non-competitive inhibition of relaxant action of bradykinin in isolated rat duodenum (Öztürk Y, unpublished results). Bradykinin induced relaxation of the rat duodenum is also dependent on extracellular calcium content.[6,40] The increase in the intracellular cAMP level by bradykinin may involve in turn phospholipid methylation, calcium flux, phospholipase $A_2$ activation and prostaglandin formation.[38] It is well known that the activity of adenylate cyclase is modulated by calcium concentration.[41] Hence, the inhibition of the bradykinin induced relaxation by trifluoperazine and verapamil shows that both calcium and calmodulin are involved in this process as well.

In conclusion, the findings obtained in the present study strongly suggest that the post-receptor events activated by bradykinin in the isolated guinea-pig ileum and rat duodenum may be different, as the inhibitory profiles of the calcium antagonists in these two tissues are not identical. Furthermore, there is a high likelihood that bradykinin $B_2$ receptors in the guinea-pig ileum and rat duodenum represent different receptor subtypes, since the apparent receptor affinity constants of bradykinin obtained from these two tissues are not of the same magnitude.

### References

1. Eisen V. Formation and functions of kinins. *Rheumatology* 1970; **3**: 103–168.
2. Regoli D. Kinins, receptors, antagonists. *Adv Exp Med Biol* 1986; **198A**: 549–558.
3. Horton EW. Human urinary kinin excretion. *Br J Pharmacol Chemother* 1959; **14**: 125–132.
4. Paegelow I, Reissman S, Vietinghoff G, Römer W, Arold H. Bradykinin action in the rat duodenum through the cyclic AMP system. *Agents & Actions* 1977; **7**: 447–451.
5. Barabé J, Park WK, Regoli D. Application of drug receptor theories to the analysis of the myotropic effects of bradykinin. *Can J Physiol Pharmacol* 1975; **53**: 345–353.
6. Faber DB, Van Der Meer C. A study of some bradykinin potentiating peptides derived from plasma proteins. *Arch Int Pharmacodyn Ther* 1973; **205**: 226–243.
7. Antonio A. The relaxing effect of bradykinin on intestinal smooth muscle. *Br J Pharmacol* 1968: **32**: 78–86.
8. Camargo A, Ferreira SH. Action of bradykinin potentiating factor (BPF) and dimercaprol (BAL) on the responses to bradykinin of isolated preparations of rat intestines. *Br J Pharmacol* 1971; **42**: 305–307.
9. Boschcov P, Antonio CM, Paiva ACM, Paiva TB, Shimuta SI. Further evidence for the existence of two receptor sites for bradykinin responsible for the diphasic effect in the rat isolated duodenum. *Br J Pharmacol* 1984; **83**: 591–600.
10. Manning DC, Vavrek R, Stewart JM, Snyder SH. Two bradykinin binding sites with picomolar affinities. *J Pharmacol Exp Ther* 1986; **237**: 504–512.
11. Burch RM, Farmer SG, Steranka LR. Bradykinin receptor antagonists. *Med Res Rev* 1990; **10**: 237–269.
12. Plevin R, Owen PJ. Multiple $B_2$ kinin receptors in mammalian tissues. *Trends Pharmacol Sci* 1988; **9**: 387–389.
13. Farmer SG, Burch RM, Meeker SA, Wilkens DE. Evidence for a pulmonary $B_3$ receptor. *Mol Pharmacol* 1989; **36**: 1–8.
14. Saha JK, Sengupta JN, Goyal RK. Effect of bradykinin on opposum esophageal longitudinal smooth muscle: evidence for novel bradykinin receptors. *J Pharmacol Exp Ther* 1990; **252**: 1012–1020.
15. Altınkurt O, Öztürk Y. Bradykinin receptors in isolated rat duodenum. *Peptides* 1990; **11**: 39–44.
16. Spedding M. Calcium antagonist subgroups. *Trends Pharmacol Sci* 1985; **6**: 109–114.
17. Levin RM, Weiss B. Specificity of the binding of trifluoperazine to the calcium dependent activator of phosphodiesterase and to a series of other calcium-binding proteins. *Biochim Biophys Acta* 1978; **540**: 197–204.
18. Altan VM, Öztürk Y, Yıldızoğlu-Arı N, Nebigil C, Lafçı D, Özçelikay AT. Insulin action on different smooth muscle preparations. *Gen Pharmacol* 1989; **20**: 529–535.
19. Ariëns EJ, Van Rossum JM. $pD_x$, $pA_x$ and $pD'_x$ values in analysis of pharmacodynamics. *Arch Int Pharmacodyn* 1957; **110**: 275–299.
20. Ariëns EJ, Simonis AM. Drug receptor interaction: interaction one or more drugs with a receptor system. In: Ariëns EJ, ed. *Molecular Pharmacology, Vol. 1.* New York: Academic Press, 1964; 119–286.
21. Finney DJ. *Statistical methods in biological assay.* London: Charles Griffin, 1978; 69–104.
22. Goldstein A. *Biostatistics: an introductory text.* New York: MacMillan, 1964; 51–60.
23. Tallarida RJ, Murray RB. *Manual of pharmacological calculation with computer programs.* New York: Springer, 1981; 1–150.
24. Rocha E, Silva M, Beraldo WT, Rosenfeld G. Bradykinin, a hypotensive and smooth muscle stimulating factor releasing from plasma globulins by snake venoms and trypsin. *Am J Physiol 1949;* **156**: 261–273.
25. Paegelow I, Reissmann S, Arold H. Charakterisierung der bradykinin wirkung am glatten muskel unter besonderer berücksichtigung der latenzzeit. *Acta Biol Med Germ* 1975; **34**: 451–461.
26. Hall DWR, Bonta IL. Effects of adrenergic blockers on the relaxation of the guinea-pig ileum by bradykinin and adrenaline. *Eur J Pharmacol* 1973; **21**: 139–146.
27. Öztürk Y, Altınkurt O, Yıldızoğlu-Arı N, Altan VM. Evaluation of the contraction time and recovery period as a parameter in the calcium antagonistic action on the $K^+$-depolarized rat duodenum. *J Pharm Pharmacol* 1990; **42**: 874–877.
28. Hertog AD, Nelemans A, Akker JVD. The multiple action of bradykinin on smooth muscle of guinea-pig taenia caeci. *Eur J Pharmacol* 1988; **151**: 357–363.
29. Portilla D, Morrison AR. Bradykinin-induced changes in inositol triphosphate mass in MDCK cells. *Biochem Biophys Res Commun* 1986; **140**: 644–649.
30. Jarrett HW, Penniston JT. Partial purification of the $Ca^{2+}$–$Mg^{2+}$-ATPase activator from human erythrocyte: its similarity to the activator of 3',5'-cyclic nucleotide phosphodiesterase. *Biochem Biophys Res Commun* 1977; **77**: 1210–1216.
31. Carafoli E, Lurini M, Benaim G. The purified calcium-pump ATPase of plasma membrane: structure–function relationships. In: Rubin RP, Weiss GB, Putney JW, eds. *Calcium in Biological Systems,* New York: Plenum Press, 1985; 265–273.
32. Huddart H, Lantham H. Calcium regulation in ileal smooth muscle. II. Interaction of sodium and magnesium in calcium counter exchange. *Gen Pharmacol* 1978; **12**: 161–168.
33. Raess BU, Gersten H. Calmodulin-stimulated plasma membrane ($Ca^{2+}$, $Mg^{2+}$)-ATPase: Inhibition by calcium channel entry blockers. *Biochem Pharmacol* 1987; **36**: 2455–2461.
34. Kim HC, Raess BU. Verapamil, diltiazem and nifedipine interactions with calmodulin stimulated ($Ca^{2+}$, $Mg^{2+}$)-ATPase. *Biochem Pharmacol* 1988; **37**: 917–921.
35. Moore KE, Kelly PH. Biochemical pharmacology of mesolimbic and mesocortical dopaminergic neurones. In: Lipton MA, DiMascio A, Killam KF, eds. *Psychopharmacology: a Generation of Progress.* New York: Raven Press, 1978; 221–234.

36. Snyder SH, Yamamura H. Antidepressants and the muscarinic acetylcholine receptor. *Arch Gen Psychiatry* 1977; **34**: 236–239.
37. Rivera-Calimlin L, Hershey L. Neuroleptic concentrations and clinical response. *Annu Rev Pharmacol Toxicol* 1984; **24**: 361–386.
38. Bareis DL, Hirata F, Manganiello V, Axelrod J. Bradykinin receptor stimulation of cAMP involves phospholipid methylation, Ca flux, phospholipase $A_2$ activation and prostaglandin formation (Abstract). *Fed Proc* 1981; **40**: 367.
39. Yeh Y-Y. Nicotinic acid reverses fasting ketosis by lowering the level of cyclic AMP. *Life Sci* 1976; **18**: 33–38.
40. Türker RK, Özer A. The effect of prostaglandin $E_1$ and bradykinin on normal and depolarized isolated duodenum of the rat. *Agents & Actions* 1970; **1**: 124–127.
41. Wiemer G, Kaiser G, Palm D. effects of $Mg^{2+}$, $Mn^{2+}$ and $Ca^{2+}$ on adenyl cyclase activity: evidence for a metallic site. *Arch Pharmacol* 1978; **303**: 145–152.

**Received 26 April 1993;**
**accepted in revised form 24 May 1993**
